# Horizontal Gene Transfer of the Secretome Drives the Evolution of Bacterial Cooperation and Virulence

**DOI:** 10.1016/j.cub.2009.08.056

**Published:** 2009-11-03

**Authors:** Teresa Nogueira, Daniel J. Rankin, Marie Touchon, François Taddei, Sam P. Brown, Eduardo P.C. Rocha

**Affiliations:** 1 Institut Pasteur, Microbial Evolutionary Genomics, CNRS, URA2171, F-75015 Paris, France; 2 UPMC Univ Paris 06, Atelier de BioInformatique, F-75005 Paris, France; 3 Escola Superior de Tecnologia da Saúde do Porto, Instituto Politécnico do Porto, 4400-330 Vila Nova de Gaia, Portugal; 4 Centro de Biologia Ambiental, Faculdade de Ciências, Universidade de Lisboa, Campo Grande, 1749-016 Lisboa, Portugal; 5 Department of Biochemistry, University of Zurich, Building Y27, Winterthurstrasse 190, CH-8057 Zurich, Switzerland; 6 Swiss Institute of Bioinformatics, Quartier Sorge Bâtiment Génopode, CH-1015 Lausanne, Switzerland; 7 Institut National de la Santé et de la Recherche Médicale, Unité 571, F-75015 Paris, France; 8 Faculty of Medicine, Paris Descartes University, F-75015 Paris, France; 9 Department of Zoology, University of Oxford, South Parks Road, Oxford OX1 3PS, UK

**Keywords:** EVOL_ECOL

## Abstract

**Background:**

Microbes engage in a remarkable array of cooperative behaviors, secreting shared proteins that are essential for foraging, shelter, microbial warfare, and virulence. These proteins are costly, rendering populations of cooperators vulnerable to exploitation by nonproducing cheaters arising by gene loss or migration. In such conditions, how can cooperation persist?

**Results:**

Our model predicts that differential gene mobility drives intragenomic variation in investment in cooperative traits. More mobile loci generate stronger among-individual genetic correlations at these loci (higher relatedness) and thereby allow the maintenance of more cooperative traits via kin selection. By analyzing 21 *Escherichia* genomes, we confirm that genes coding for secreted proteins—the secretome—are very frequently lost and gained and are associated with mobile elements. We show that homologs of the secretome are overrepresented among human gut metagenomics samples, consistent with increased relatedness at secretome loci across multiple species. The biosynthetic cost of secreted proteins is shown to be under intense selective pressure, even more than for highly expressed proteins, consistent with a cost of cooperation driving social dilemmas. Finally, we demonstrate that mobile elements are in conflict with their chromosomal hosts over the chimeric ensemble's social strategy, with mobile elements enforcing cooperation on their otherwise selfish hosts via the cotransfer of secretome genes with “mafia strategy” addictive systems (toxin-antitoxin and restriction-modification).

**Conclusion:**

Our analysis matches the predictions of our model suggesting that horizontal transfer promotes cooperation, as transmission increases local genetic relatedness at mobile loci and enforces cooperation on the resident genes. As a consequence, horizontal transfer promoted by agents such as plasmids, phages, or integrons drives microbial cooperation.

## Introduction

Humans live in intimate mutualism with many microbes that are important for nutrient uptake and to stabilize niches prone to invasion by pathogens. The human gastrointestinal tract records the highest cell densities for any known ecosystem with ∼10^14^ individuals from more than 500 species that cooperate and compete while interacting with the host [1]. Among them, *Escherichia coli*, the workhorse of molecular biology, is a major colonizer of the human gut where it establishes associations that are most frequently commensal but that can in some cases be highly virulent. The genome of *E. coli* shows a remarkable variability of its gene repertoire [2–4]. *E. coli* genomes have an average of ∼4600 protein-coding-genes of which many are strain specific and less than half are ubiquitous (the core genome) [5]. This dynamic gives the species great adaptability and ecological diversity. The outcome of its interaction with the host depends on its ability to adhere to cell surfaces, colonize tissues, and produce metabolites and on its interplay with other bacteria. Many of the genes associated with ecological interactions are present in the genomes of both commensal and pathogenic strains, suggesting that this species, like many others, should be regarded as a complex of strains oscillating between commensalism and pathogenicity [5, 6].

Many interactions between *E. coli* and other microbes, eukaryotes, or abiotic factors depend on secreted or outer-envelope-exposed molecules (e.g., [7, 8]). These molecules, although presumably costly to produce, generate a range of benefits to any neighboring bacteria that are suitably equipped to profit from the expression of these molecules. For example, the molecules may scavenge for scarce resources (e.g., siderophores), aid in the construction of biofilms (e.g., adhesive polymers), kill competing lineages (e.g., bacteriocins), or aid direct exploitation of a host (e.g., shiga toxins). Diffusion rates will have an important role in evolution of the trait. Thus, outer membrane proteins are likely to benefit the most neighboring cells, whereas secreted proteins may diffuse farther in the environment. The shared rewards generated by these surface and secreted molecules ensures that their producers are prone to exploitation by nonproducer “cheaters” that prosper at the expense of more cooperative individuals. The above traits are all cooperative from the perspective of individual cells that are equipped to benefit from the shared molecules. For example, bacteriocin production is aimed at eliminating competitors in the community (and can be viewed as spiteful), but it is still a cooperative trait among the individuals carrying the appropriate production and resistance genes [9]. A thorough discussion of logical identities between altruism and spite can be found elsewhere [10]. The recognition that microbial-shared (surface or secreted) molecules are public goods, vulnerable to exploitation by cheaters, brings to the fore the need to understand social dilemmas raised by microbial cooperation [11, 12].

**Figure 1:**
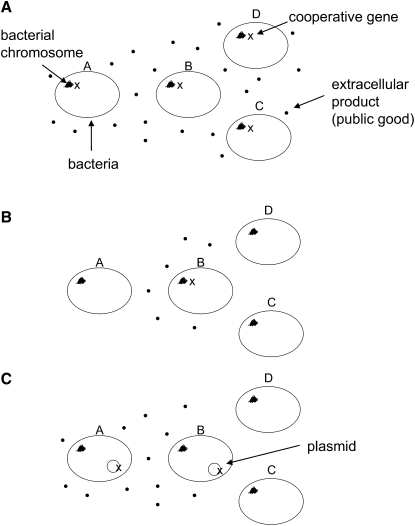
The Influence of Population Structure and Relatedness on Bacterial Social Traits in a Subpopulation A cooperative gene coding for a public good is represented by “x” and can be carried either by the chromosome (A and B) or a plasmid (C). (A) A small bacterial population where all individuals have the cooperative gene on the chromosome. (B) Only one individual (B) posseses the gene and produces the public good. A, C, and D benefit from B's actions. (C) The cooperative gene is carried on the plasmid so individuals A and B both produce the trait. A and B will now have a high genetic relatedness (*r* > 0) because relatedness is defined as the probability that two individuals bear the same gene. Thus relatedness is influenced by the rate at which the plasmid spreads through the subpopulation. The greater the plasmid infection rate, the more “related” the hosts will be because more individuals within the subpopulation will share the same genes, albeit on the plasmid. This illustrates that it is the focal locus that ultimately matters when dealing with social dilemmas such as public good production.

The evolution of cooperation can be favored by both nepotism and self-interest [12]. Cooperation may be self-interested if it directly benefits the actor. More extreme forms of altruistic cooperation may also be evolutionarily favored when they differentially help recipients who are statistically more likely to share the altruistic gene, i.e., self-interest returns at the level of the gene. Yet, when genomes are highly dynamic and environments very diverse, such as in microbial populations in the human gut, how can cooperation persist in the face of cheaters constantly arising by gene loss or migration? Initial theoretical work has suggested that the invasion of cheaters in a population of cooperators would be prevented if the social trait were coded in conjugative plasmids [13], because cheaters created by plasmid loss would (re-)acquire the trait by reinfection with the plasmid; sociality would be restored because of the infectivity of the social trait. A key assumption of this model is that all plasmids carry the cooperative trait, so any act of infection will also increase cooperation. But what of the social dilemma between cooperative and cheating traits, played out at the level of the mobile element? In principle, cheaters created by loss of the cooperative gene but not of the whole plasmid will still be able to invade because they are both infectious and social cheaters, benefiting from the cooperative actions of their neighbors (Figure 1). In order to understand the fate of cooperative traits in this broader strategy set (allowing for both cooperative and noncooperative mobile elements), we must return to the basic principles of social evolution theory, and ask—what are the inclusive fitness consequences of carrying a cooperative trait, as a function of gene mobility?

## Results

**Figure 2:**
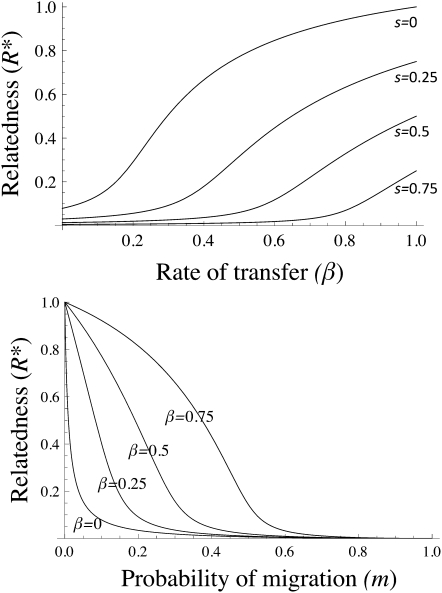
Effect of Migration and Transfer Rates on Relatedness Top: Effect of the probability of horizontal gene transfer on relatedness within a patch under different gene loss probabilities (*s* = 0, *s* = 0.25, *s* = 0.5, *s* = 0.75), where *m* = 0.1 and n = 50. Bottom: Effect of the migration rate *m* on relatedness for different degrees of horizontal gene transfer (β = 0, β = 0.25, β = 0.5, β = 0.75), where *s* = 0 and n = 50.

A general result of social evolutionary theory is that an altruistic gene, which confers a benefit *b* on another individual at a cost *c* to an actor can spread in a population if *Rb* > *c*, where *R* is the genetic relatedness between two individuals and is measured with respect to the locus controlling the behavior in question [14]. Our model (fully described in Supplemental Data available online) uses a standard recursion equation for relatedness in a patch-structured population assuming a basic life-cycle, where individuals reproduce, interact, and migrate, and finally population regulation occurs [15]. We extend this recursion to allow for horizontal gene transfer based on the formalism of unbiased horizontal transmission of cultural traits [16] (where the change in frequency of the horizontally transferred traits depends purely on its frequency in the local population and not on any allelic value). The probability that two individuals carrying distinct alleles become identical at the focal locus in one time-step will depend on gene mobility β at this locus and the within-patch homogeneity at this locus, *R*(*t*), for a patch of a given size (*N*). The probability that two individuals carrying identical alleles remain identical in one time-step depends on the rate of gene loss (*s*). When both within-patch gene mobility and segregation loss tend to zero (*β*→0 and *s*→0), the recursion equation converges to an equilibrium at $R*={\left(1-m\right)}^{2}/(N-{\left(1-m\right)}^{2}\left(N-1\right))$, where *m* is the among-patch migration rate, capturing relatedness (or *F_st_*) as a function of deme size and migration, under purely vertical transmission. Incorporating genome dynamics into our calculation of relatedness within patches shows that horizontal transfer increases relatedness, whereas gene loss reduces it (Figure 2). Because increased local relatedness favors cooperation [14, 17], we conclude that horizontally transferred genes will be more likely to code for cooperative traits than those that are less infectiously mobile.

**Figure 3:**
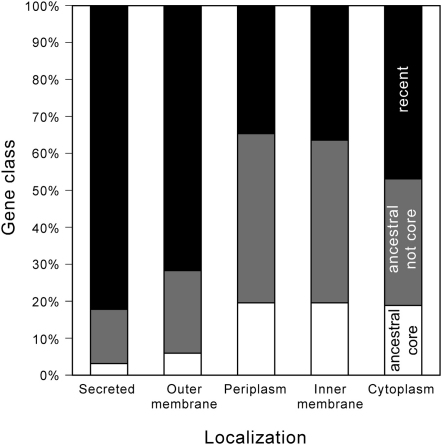
Distribution of Core, Ancestral, and Recent Genes among the Localization Classes The differences are highly significant (χ^2^ test, p < 0.0001).

To test our model, we inferred protein localization in 21 genomes. We analyzed 20 *E. coli* genomes and their plasmids and also *E. fergusonii*, which is the closest outgroup (Table S1). Gene dynamics in *E. coli* is high enough to change significantly even at short time spans, providing opportunity for social traits to be gained, exchanged, and lost. To avoid overrepresenting gene families that have endured extensive recent duplication, e.g., transposable elements, we put together very closely related (>80% protein sequence identity) families of orthologs. Proteins within a family, henceforth named equivalogs, are assumed to have similar functions and localization. We inferred protein localization via pSORTb [18] and secretion by type 3 secretion systems (T3SS) via sequence similarity to known effectors [19]. By using a conservative approach (see Experimental Procedures), we inferred the localization of ∼5,700 families for a total of ∼59,000 proteins (Table 1). This corresponds to 43% of all families of equivalogs and 58% of all genes. As expected, the majority of proteins are cytoplasmic (61.3%), many are associated with the inner membrane (25.5%), and proteins localized in the periplasm (4%), in the outer membrane (5.6%), and secreted (3.6%) are rarer. Secreted and outer membrane proteins are much less frequently ubiquitous, i.e., present in the core genome, or ancestral, i.e., present in the last common ancestor of *E. coli*, than expected (both p < 0.0001, χ^2^ test). Inner membrane, periplasmic, and cytoplasmic localizations have similar fractions of genes in the core genome (∼24%), whereas only 6% of outer membrane proteins and 3% of secreted proteins are in the core genome (Figure 3). The frequency with which external proteins are gained and lost is thus consistent with our prediction that cooperative traits are mobile (β > 0). This pattern of frequent gene gain and loss is entirely consistent with both theory and experimental evolution studies on microbial social traits, which have repeatedly revealed how readily selection on cooperative traits can be reversed as a function of small changes to population structure (e.g., [20, 21]).

**Figure 4:**
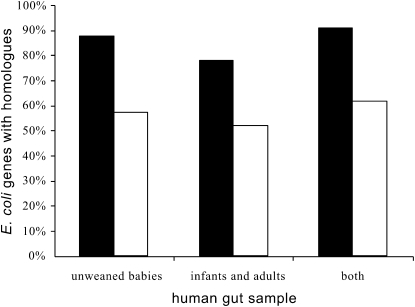
Percentage of Nonancestral *E. coli* Genes with Homologs in Human Gut Metagenome The bars correspond to secreted and outer membrane proteins (black bars) and other localizations (white bars), when separating the set of unweaned babies from the sets containing other individuals and when including all data [23].

Our hypothesis depends on the existence of genes coding for public goods, especially for nonancestral secreted and outer membrane proteins, in *E. coli*'s environment. Because many horizontally transferred genes lack homologs in the current databases [22], we searched for homologs of *E. coli* proteins from the secretome or from other localization in a large (427,289 proteins) human gut metagenome sample [23]. We first checked that the secretome genes had family sizes not significantly different from the remaining localizations (p > 0.05, for both Student's t and Wilcoxon tests). We then observed that in spite of similar family sizes, many more genes of the secretome (91%) had homologs in the data set than for the other localizations (∼60%, p < 0.0001 in all cases, Figure 4). Some of the hits might correspond to *E. coli* cells in the sample. Our model assumes that secretome genes are present in other bacteria in the environment but makes no specific prediction about these genes occurring in the same or different species. Nevertheless, we tried to further detail this point by estimating the number of *E. coli* proteins in the metagenomic sample. For this, we separated the adult and infant data sets, where *E. coli* proportions are ∼10^−5^/bacteria, from the unweaned baby data set, where *E. coli* is more abundant. The former contains ∼340,000 proteins leading to an expected number of 34 *E. coli* proteins present in the metagenomic sample, of which three are secreted or outer membrane associated. Thus, when we find that most of the secretome has homologs in the environment, this almost never corresponds to *E. coli* genes present in the metagenomics sample, but instead to hits to genes in other species genomes. This suggests that cooperation by mobilization of public goods crosses species barriers establishing relatedness between previously unrelated bacteria.

Many of the proteins we predict as secreted are annotated as virulence factors, consistent with the view that microbial virulence is driven by cooperative bacterial traits [24, 25]. Yet, “virulence factor” labels often mask broader social traits. For example, we identified a secreted flagellin in enteroaggregative strains. Flagellins are involved in the inflammation process [26], but they are also involved in immunomodulation by probiotic *E. coli* strains [27]. Similarly, the toxins secreted by the enterohemorrhagic strain O157:H7, which are mobilized by a prophage, have no effect in cattle (where the strain is a commensal), but can be highly virulent in humans [28]. This shows the thin line between mutualism and antagonism and suggests a broader, and potentially multivalent, role of secreted proteins in social interactions. We found four times less T3SS effectors in commensals than in pathogens (p < 0.0001, χ^2^), in line with the available evidence for the role of T3SS in virulence but not in commensalism in *E. coli* [19]. Excluding the small set of T3SS effectors, there is no significant difference in secretome size between commensals and pathogens (both p > 0.4, χ^2^). Secreted and outer membrane proteins perform a variety of functions that are not necessarily related with virulence, even if their role in virulence is well described. Interestingly, *Shigella* stands out as having three times less secreted proteins than expected given genome size (p < 0.01, χ^2^). *Shigella* thrives within eukaryotic cells where they have little opportunity for social interactions. The ensuing lower rates of transfer might then lead to loss of cooperative traits in these strains, in spite of the strain's virulence, and in agreement with our model. These results highlight the ubiquity of microbial social life: bacteria are social engineers, and this engineering poses social dilemmas that affect but are not limited to virulence.

**Figure 5:**
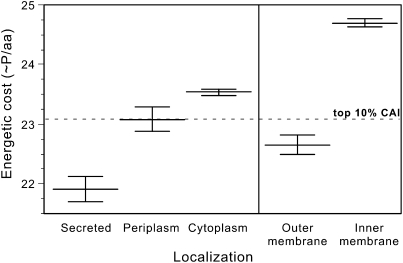
Biosynthetic Energetic Cost per Residue in Proteins The large horizontal line indicates the average and the two small lines indicate its 95% interval of confidence. The dashed line indicates the cost of the 10% highest expressed genes via the codon adaptation index (see Figure S4 for details). The distribution is significantly different from the expected (χ^2^ test, p < 0.0001).

Secreted proteins are metabolically costly for the producer, they are poorly or not recycled, and because they are public goods posing social dilemmas, they are potentially rewarding for the cheaters. They should therefore present traces of selection for the use of biosynthetically inexpensive amino acids. The *E. coli* biosynthetic cost of each amino acid from basic precursors was used to compute an average cost for each residue in each protein (as in [29]). We found that proteins were cheaper when they were exterior (Figure 5). This difference remains unchanged when controlling for gene expression levels (Figure S1) or ancestrality (Figure S2), and is even higher when controlling for G+C content (Figure S3). Membrane proteins were compared separately because they have peculiar structures and cannot meaningfully be compared with the other proteins in terms of amino acid composition. Inner membrane proteins are more expensive than outer membrane proteins (p < 0.0001, Wilcoxon test). Cytoplasmic proteins are more expensive than periplasmic and these in turn are more expensive than secreted proteins, which are the least expensive of all proteins (p < 0.0001, Wilcoxon test). This suggests a selection gradient for lower cost in the most external proteins in both membrane-associated and -nonassociated proteins. Hence, secreted proteins endure the strongest selection for low biosynthetic cost, consistent with their potential exposure to social exploitation by nonproducer cheaters.

In fact, secreted and outer membrane proteins are cheaper than highly expressed proteins (Figure 5), suggesting that localization is more important than expressiveness in leading to selection for inexpensive amino acids. Indeed, although the linear regression of expression levels measured by the codon adaptation index explains less than 9% of the variance in protein cost (Figure S1), the ANOVA of the protein localization on protein cost accounts for 18%. The strong association between protein cost and localization shows that the costs of public good provision can be partly alleviated by selection of less costly amino acids.

**Figure 6:**
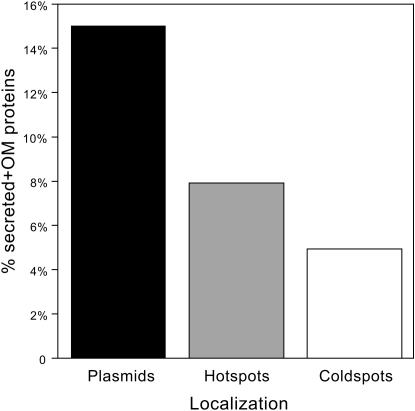
Positioning of Genes Coding for Secreted and Outer Membrane Proteins Frequency of genes that are not in the core genome within three types of genome positioning: plasmids, chromosomal hotspots, and other locations in the genome. The differences are highly significant (χ^2^ test, p < 0.0001).

Our model suggests that relatedness increases because of horizontal gene transfer. In consequence, mobile genes should be more likely to offset the costs of investing in a cooperative trait via greater inclusive fitness benefits. These results are expected to be applicable to mobile elements in general, even integrative ones, as long as they are not strongly deleterious. Therefore, our prediction is that cooperative traits should be preferably coded in the most mobilizable regions of genomes. Some regions of the *E. coli* genome constitute transfer hotspots whereas others are very stable. Indeed, 133 hotspots accumulate more than 70% of all variable genes in the chromosomes of the 20 strains analyzed in our study [5]. Therefore, genes are highly mobile if they are in plasmids, very mobile if they are in hotpots, and weakly mobile if they are in the remaining genome (coldspots). Naturally, for this analysis we removed all nonvolatile genes, i.e., the core genome. We then analyzed the position in the chromosome of genes coding for proteins with inferred localization. Secreted and outer membrane protein coding genes are more frequent in plasmids (15% of all genes) than in hotspots (8%) and than in coldspots (5%), where they are three times less frequent than in plasmids (p < 0.0001, χ^2^ test) (Figure 6). The effect is nearly two times stronger in secreted than in outer membrane proteins. This confirms the prediction of the model that secreted and outer membrane proteins are more often located in more mobile regions.

**Figure 7:**
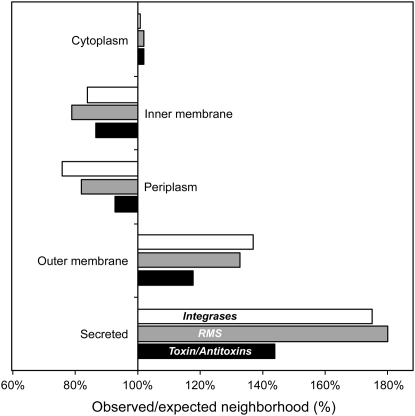
Functions in the Neighborhood of Genes Coding for Localized Proteins Observed/expected co-occurrence of genes that are not in the core genome coding for proteins localized in different cellular regions with integrases, restriction/modification systems, and toxin/antitoxin systems. The distributions are significantly different from the expected values for all three types of genes (χ^2^ test, p < 0.0001).

Plasmids contain 16 times fewer genes than hotspots and 7 times fewer than coldspots. Therefore, even if relatively fewer genes coding for external proteins are in chromosomes, these still account for a large fraction of the secretome. Both in hotspots and in coldspots, some elements are more prone to mobilization because they are colocalized with integrases that facilitate the integration of such regions in other genomes. We found a very high colocalization of integrases with secreted proteins, and, to a lesser extent, with outer membrane proteins (p < 0.0001, Wilcoxon test; Figure 7). This further supports our model expectation that cooperative traits are encoded in regions that are highly mobile.

The frequency of conjugation depends on the donor but very little on the recipient bacterium [30, 31], favoring the spread of cooperative genes, and consequently the imposition of cooperator phenotypes on what were previously defector cells [13]. Many species maintain cooperation via strategies of enforcement (e.g., sanctions, policing, punishment [32, 33]). Whereas most enforcement strategies entail death or isolation for defectors, the transmission of mobile genes acts to enforce cooperation by reprogramming defectors. However, the volatility of mobile genetic elements may lead to a high rate of gene deletion with subsequent production of new cheaters (represented by the segregation rate *s* in our model). The maintenance of cooperation would be further facilitated if social traits were encoded along with stabilizing genetic elements. Type II restriction-modification and toxin-antitoxin systems have been shown experimentally to result in the stabilization of plasmids but also chromosomally encoded laterally transferred genes [34–36]. We therefore analyzed how these genes colocalized in the chromosome with genes not in the core genome and for which we could infer protein localization. We found a highly significant copositioning of these stabilizing elements with the genes coding for secreted proteins, and, to a lesser extent, with genes coding for outer membrane proteins (p < 0.0001, Wilcoxon test) (Figure 7). The copositioning of mobile cooperative genes with addiction and restriction-modification complexes will act to further enhance the enforcement of cooperation on the chimeric bacterial individual, by punishing the loss of the cooperative trait.

## Discussion

The average human body carries asymptomatically more than 10^8^ *E. coli* cells [37]. Yet, infections by strains of *E. coli* result in nearly one billion cases of diarrhea per year, leading to more than a million human deaths [38]. Shifts between mutualism and parasitism are largely the result of complex social interactions among microbes and the human host [1]. Besides enterobacteria, they also concern bacteria notorious for having high rates of genetic transfer such as *Neisseria*, *Streptococcus*, *Staphylococcus*, *Helicobacter*, or *Bacteroides*. Most strains in these genera are commensal and are carried by a large percentage of the population, but some strains in some circumstances can be deadly pathogens. The disturbance in the social network of commensals caused by the loss of cooperative traits and the subsequent demographic depression may open the niche for other bacteria, eventually pathogens. Inversely, the fitness loss associated with the disruption of cooperative behavior of pathogens may facilitate therapy [39], e.g., by weakening their invasive potential. It is therefore important to know how networks of cooperators can be stably maintained or disrupted for public health reasons [40]. Our results suggest that this may be of general utility to manipulate microbial social behavior.

The statistical association between gene mobility and engagement with the secretome is open to additional explanations. Some outer membrane proteins in bacterial pathogens and commensals are recognized by the immune system, by grazing protozoa, or by phages and are thus under diversifying or frequency-dependent selection [41–43]. In the best-studied cases, this leads to the selection of simple sequence repeats that generate variability at the promoter or at the protein sequence level [44], to selection for gene multiplicity for gene conversion between homologous sequences [45], or to signs of positive selection [46]. In the vast majority of cases, this affects outer membrane proteins. Although diversifying selection surely acts upon some *E. coli* outer membrane proteins, it is unlikely to fully explain the patterns we observe in Figure 3 because the effect is especially strong among secreted, not outer membrane, proteins. Also, *E. coli* codes for few simple sequence repeats [47], few large repeats putatively involved in gene conversion [48], and few genes under strong positive selection [43], suggesting that diversifying selection may not be so important in this species. Finally, although immune challenges are more important for pathogens than for commensals [49], we found no association between pathogenicity and secretome size. In fact, the highly virulent *Shigella* strains have fewer outer membrane proteins than the remaining genomes. Further work will be necessary to disentangle the effects of different types of selection on the patterns found in Figure 3.

More importantly, even if all genes not in the core genome were under other forms of diversifying selection, the secretome would still face social dilemmas. The benefits of the secretome (e.g., nutrient acquisition) are indeed likely to be environment specific, whereas the benefits of the core genome (replication, repair, etc.) are more likely to be environment independent. The fluctuating rewards of investment in the secretome may therefore promote their flexible gain and loss as part of an accessory genome (Figure 5) [50]. Yet, within this accessory genome putatively under the action of diversifying, frequency-dependent, or weak selection, we systematically find deviations in the sense predicted by our model, i.e., among noncore genes, the ones coding for the secretome, which are the ones most facing social dilemmas, are more frequently in the environment, in mobile elements, and associated with enforcement factors. Although the inconsistent and accessory nature of many secretome traits may contribute to their emergence on mobile elements, their maintenance will depend strongly on their resilience in the face of social cheaters—because whenever they generate rewards, these rewards will by their extracellular nature be shared. Therefore, the social forces (shaped by gene mobility and extracellular benefits) outlined in our study will remain central to any understanding of the maintenance of social traits on mobile genes.

Given the highly promiscuous nature of horizontal gene transfer in prokaryotes, mobile genetic elements may bring inclusive fitness benefits to cooperation among species: by helping heterospecifics that also contain the element, the element (e.g., a plasmid) benefits a copy of itself. Plasmids and conjugative transposons are often highly promiscuous and their rates of exchange can be as high or higher between species than among conspecifics [30]. Some plasmids have very broad ranges and can autonomously transfer into hosts that have diverged billions of years ago [51, 52]. For example, transfer ranges of IncP plasmids encompass *Proteobacteria*, *Firmicutes*, *Bacteroides*, and yeasts [53], thus potentially all major microbial constituents of the human gut. Conjugation itself promotes biofilms that may further facilitate horizontal transfer [54]. Our observation that social traits coded in *E. coli* are frequent in the environment (Figure 4) and overrepresented in its mobile elements (Figure 6) opens the possibility of kin selection (defined as the process by which inclusive fitness is maximized [32]) among these organisms, broadening the conceptual framework for the evolutionary study of between-species mutualisms [55]. As a result, our analysis does not simply concern cooperation between a few strains of *E. coli*, but should be interpreted in a more global view of cooperation between microbial species, related via their shared mobile elements.

Our model relies on frequent transfer between microbes for the maintenance of cooperation. Further work will be necessary to determine what are the minimal rates of transfer to maintain cooperation in *E. coli*'s primary or secondary habitats. One can suppose two extreme values for the time frame of transfer in the primary habitat. Dominant *E. coli* strains persist for months in the host [56], which is an upper bound, and intestinal transit times average ∼60 hr in humans [57], which is a lower bound. Conjugation and transduction occur both in the lab and in vivo at rates compatible with the lower bound given by intestinal transit [58, 59].

Our results bring new light to the role of vehicles of lateral transfer, such as plasmids, integrative conjugative elements, or temperate phages, and help explain why they are so prevalent in bacterial populations [60, 61]. Temperate phages code for a range of social traits, e.g., toxins [28], and have been demonstrated to generate higher relatedness coefficients at prophage loci than at their host chromosomes: competition experiments between prophage-carrying (lysogenic) and nonlysogenic bacteria resulted in the maintenance of host chromosomal diversity, apart from monomorphism at the prophage loci [62]. Integrons, which are often coded in plasmids, code for many proteins with peptide signals, thus probably secreted [36]. These and other integrative highly dynamic gene-capture systems may therefore constitute powerful generators of microbial social networks. If plasmids are major players in the dynamics of social interactions, as our data suggest, it is essential to further characterize the diversity of genetic information they code for.

Cooperation mediated by secreted proteins is metabolically costly. Protein biosynthesis is among the costliest of cellular processes because of both the costs of the raw materials (amino acids) and the costs of synthesis (gene expression and the production of the necessary machinery) [63]. Recent data suggest that the process is more costly than the product [64], among other reasons because amino acids can be recycled after proteolysis. Yet, secreted proteins cannot be easily recycled, and possibly so cannot the exterior domains of outer membrane proteins, making them more expensive. As a result, we find evidence of selection for biosynthetically less expensive amino acids in these proteins that partly alleviate the burden of producing public goods. Naturally, we cannot exclude that an unknown biophysical effect leads to the selection of biosynthetically inexpensive amino acids in the exact same way as predicted by our model. The extent of selection for biosynthetic cost might seem surprising, because accessory genes are traditionally supposed to be under weak selection, yet we find evidence of selection for their amino acid composition. The cost of secreted and outer membrane proteins might be under stronger selection as a result of both their limited recyclability and their involvement in cooperative processes, vulnerable to exploitation by nonproducer cheats.

If cooperation is costly, how can it arise and be maintained in bacterial populations enduring migration and gene deletions resulting in new cheaters? We find, on theoretical grounds, that the mobilization of genes producing public goods favors the maintenance of cooperation by increasing relatedness at the mobile loci. It is well established that relatedness favors investments in cooperation [17, 32, 65]. By increasing relatedness (at mobile loci) between interacting individuals, horizontal transfer should therefore favor cooperation, coded by the mobile loci. However, the imposition of a social trait on a distinct recipient genetic background raises the potential for intragenomic conflict, resulting ultimately from the intragenomic variation in mobility. Whereas the most mobile genes will gain a high inclusive fitness benefit (defined by *Rb-c*) from providing benefits to neighboring bacteria (because they are more likely to carry a copy of the same gene), other more static genes may experience a loss of inclusive fitness from aid to the same bacterial neighbors—because they are less likely to be related at nonmobile loci—and may therefore be selected to inhibit the uptake or carriage of more mobile—and social—genes. We find that mobile genes act to enforce cooperation [32, 33] on their chromosomal hosts via the cotransfer of cooperative genes with addictive systems that together impose a “mafia” [66, 67] ultimatum to their host of “cooperation or death.” Thus we find that cooperation among bacterial individuals, driven by mobile genetic elements, is in turn the cause of conflict within these individuals, between differentially mobile genes.

## Experimental Procedures

### Definition of the Core Genome and Gene Ancestry

The preliminary core and pan-genome were computed as described elsewhere [5]. Because paralogs with more than 80% similarity and similar size are likely to have very similar functions and localizations, we put such families together into families of equivalogs. By using the pattern of presence/absence of equivalogs, we inferred their acquisition/loss in the history of the *E. coli* species, by using maximum likelihood, with the package *ape* in R [68]. We redid all the analysis with inferences of ancestrality based on orthologs instead of equivalogs, with similar results (data not shown). The final core genome contains families with equivalogs in all genomes. All genes of the core genome are inferred to be present in the ancestor genome.

### Definition of Homology

Protein sequences of integrases and toxin/antitoxin systems were fetched from Swissprot (http://www.expasy.ch/sprot/), type 2 restriction/modification systems from Rebase (http://rebase.neb.com/), and T3SS effectors from [19]. Homologs were searched in the genome with BlastP and selected when E-value < 10^−5^. We considered two genes as homologs if they aligned through more than 80% of their sequence and were >40% similar.

### Prediction of Cellular Localization

Proteins specifically targeted for secretion can be identified from their sequences [18, 69]. We initially used more than 10 programs to infer protein localization. Many of these programs provide putative localizations even when their likelihoods are low. We wished to remain conservative in our analysis and use a reliable data set of proteins and therefore excluded them. The other programs often gave similar results. Because pSORTb has been evaluated as one of the best performing programs, we used it extensively [18]. The results of several tests of the pSORTb protein localization prediction program [70] were integrated: ECSVM-, the Gram-negative version of the support vector machine trained to identify extra cellular proteins; OMSVM-, the support vector machine trained to identify outer membrane proteins for Gram-negative bacteria; PPSVM-, the support vector machine trained to identify periplasmic proteins for Gram-negative bacteria; CMSVM-, the Gram-negative version of the support vector machine trained to identify cytoplasmic membrane proteins; HMMTOP-, which predicts transmembrane helices within the sequence (where the presence of three or more transmembrane helices are predicted to belong to the inner membrane); CytoSVM-, the Gram-negative version of the support vector machine trained to identify cytoplasmic proteins; and the Final prediction. We also used the TMHMM program to identify transmembrane helices in integral membrane proteins [71] and kept proteins with at least three transmembrane helices. Type 3 secretion systems (T3SS) have been thoroughly studied for their role in transport of effectors allowing the manipulation of eukaryotic cells by pathogenic *E. coli*. T3SS effectors can only be identified by sequence similarity with known effectors. We therefore complemented our catalog of secreted proteins by searching for proteins similar to a recently published set of known T3SS effectors in proteobacteria [19].

### Metagenomic Data

The contigs from 13 healthy humans gut microbiomes of the Human Metagenome Consortium Japan [23] (HMGJ; http://www.metagenome.jp/) were retrieved from GenBank. Genes were identified with the program getorf from the EMBOSS package (http://emboss.sourceforge.net/) [72].

## Figures and Tables

**Table 1:** Cellular Localization Prediction of the Pangenome Proteins

	Programs	Families of Equivalogs	Percent of the Pangenome	Number of Genes
T3SS^a^	BlastP	131	0.9%	738
Secreted	Psortb	150	1.1%	634
Outer membrane	Psortb	331	2.5%	2,017
Periplasm	Psortb	240	1.8%	3,014
Inner membrane	Psortb, TMHMM	1,511	11.4%	17,690
Cytoplasm	Psortb	3,612	27.4%	36,917

a Some T3SS effectors are also predicted to be localized elsewhere by PsortB.

## Supplemental Data

Document S1. Supplemental Experimental Procedures, Three Figures, and One Table
