# Supplementary material for: Horizontal Gene Transfer of the Secretome Drives the Evolution of Bacterial Cooperation and Virulence
Source: Curr Biol. 2009 Nov 3;19(20):1683–91. doi: 10.1016/j.cub.2009.08.056 (PMC2773837; doi:10.1016/j.cub.2009.08.056)
Supplement: Document S1. Supplemental Experimental Procedures, Three Figures, and One Table [file mmc1.pdf]

Current Biology, Volume *19*

Supplemental Data

Horizontal Gene Transfer of the Secretome Drives the Evolution of Bacterial Cooperation and Virulence

Teresa Nogueira, Daniel J. Rankin, Marie Touchon, François Taddei, Sam P. Brown, and Eduardo P.C. Rocha

1. Detailed model

A general result of social evolutionary theory is that an altruistic gene, which confers a benefit *b* on another individual, at a cost *c* to an actor can spread in a population if $Rb>c$, where *R* is the genetic relatedness between two individuals [1-4]. The parameter *R* represents the genetic covariance between the two individuals, and is measured with respect to the locus in question. Genetic relatedness generally depends on the demography of a species and their ability to discriminate kin from non-kin, and will be enhanced if individuals do not disperse from where they are born (but note that while population viscosity can raise relatedness, it can also diminish the net benefits (b) of indiscriminate cooperation due to increased competition among relatives) and are able to target rewards to differentially towards kin [1, 2, 5, 6]. In such cases neighbours will be more likely to be related and *R* will be high.

We begin our analysis with a standard recursion equation for relatedness in a patch-structured population. We assume a basic life-cycle, where individuals reproduce, interact, migrate and finally population regulation occurs. We define *R* as the probability that two randomly picked individuals within a patch carry identical alleles at the focal locus. We assume that the population is subdivided into an infinite number of patches of size *N*, the probability that a given bacterial individual migrates to another patch in a given time interval is *m* and that selection is weak. Together these assumptions yield the recursion $R(t+1)=(1-m)^2\left(\frac{1}{N}+\frac{N-1}{N}R(t)\right)$. Here $(1\text{-}m)^2$ is the probability that two random individuals will remain in a patch during a given time-interval and $1/N$ is the probability that the two non-migrant individuals stem from the same parent in the previous timestep (For more details, see [4]). We now extend this recursion to allow for horizontal gene transfer. We can expect that horizontal gene transfer (e.g via plasmid conjugation) will affect relatedness within a patch, either through the plasmid infecting other individuals (and thus increasing relatedness) or through plasmid loss (due to segregation)

$$R(t+1) = (1-m)^2 \left(p_K \frac{1}{N} + \frac{N-1}{N} \left(p_K R(t) + p_G (1 - R(t)) \right) \right) + p_G \left(1 - (1-m)^2 \right) \qquad (1)$$

This equation is based on the unbiased horizontal transmission of cultural traits [7]. Here we allow for both the loss and gain of identity in state at the loci of interest, as a result of gene loss and within-patch gene transmission. p_K is the probability that two individuals carrying identical alleles at the focal locus remain identical in the next time-step and p_G is the probability that two individuals carrying distinct alleles become identical in the next time-step. Again, we assume that selection is weak, a standard assumption in models of social evolution [8].

The probability that two individuals carrying distinct alleles become identical at the focal locus in the next time-step (p_G) will depend on gene mobility β at this locus, and the within-patch diversity at this locus, $R(t)$. Here we assume that $p_G = \beta R(t)$, where β can be viewed for plasmids as the probability that conjugation will occur. The probability that two individuals carrying identical alleles remain identical in the next time-step (p_G) will depend on the potential for gene loss due to segregation, such that $p_K = 1 - s$, where s is the probability that neither of the pair segregate the gene. There are other ways in which horizontal transmission p_G may be modelled [9] but these do not change the qualitative nature of our results (not shown).

At the limit where both within-patch gene mobility and segregation loss tend to zero ($\beta \to 0$ and $s \to 0$), the recursion equation (1) converges to an equilibrium at $R^* = \frac{(1-m)^2}{N - (1-m)^2 (N-1)}$, capturing relatedness (or Fst) as a function of deme size and migration, under purely vertical transmission [4]. At the limit when $N \to \infty$ and $m \to 0$ (i.e. a very large patch, with no migration), equation (1) converges to $R^* = 1 - s/\beta$, which is the proportion of cells infected by a plasmid within a very large, well-mixed patch (a basic epidemiological result for the prevalence of infected individuals as a function of transmission and clearance [10]). Under these conditions, segregation reduces relatedness, while transmission increases relatedness. Additionally, if $s=0$ and $m=0$ (i.e. there is no migration between patches, and plasmids are never lost) then relatedness converges to 1, as all cells eventually become infected with the plasmid.

Figure 2 shows how horizontal transfer affects relatedness within a patch, when R is at equilibrium (R^*). Incorporating horizontal transfer into our calculation of relatedness shows that horizontal transfer of plasmids (plasmid infection) increases plasmid loci relatedness within patches, while segregation reduces relatedness. As increased local relatedness favours cooperation [1-3, 11], we conclude that horizontally-transferred genes will be more likely to code for cooperative traits than those that are less infectiously mobile. The effect of gene mobility on relatedness is illustrated in

figure 2, which shows the standard result that migration decreases relatedness within a patch (e.g. [6]): allowing for horizontal gene transfer greatly increases local relatedness. Therefore, we expect mobile loci to experience higher relatedness than more static loci, and therefore selection will favour infectious plasmids carrying cooperative traits. Our model suggests that relatedness does indeed increase due to horizontal gene transfer, and that this should be enough to offset the costs of investing in a social trait. While based on the biology of conjugative plasmids these results are expected to be applicable to mobile elements in general, including elements integrating in the chromosome. As such, our prediction is that social genes should be preferably coded in the most mobilisable regions of genomes.

Throughout this model we used the example of conjugative plasmids as they are well-known self-transmissible genetic elements that were also considered in previous works [12]. Our result is expected to be applicable to many other mobile elements such as integrative conjugative elements and mobilizable plasmids. In general we predict that the most mobile elements should be the ones carrying more cooperative traits. However, this may not be applicable to virulent phages, as they typically kill their hosts, which would violate our assumption of weak selection. It is an open question if the gains in relatedness are enough to offset the risk of cell death by temperate phages, knowing that prophages can be highly efficient weapons of niche invasion by lysogenic bacteria [13].

2. Supplementary table

Table S1. Number of proteins per genome. The pathogenicity character was taken from [14]. Genomes without sequenced plasmids are indicated as "0" under "plasmidic".

Strain	Pathogenicty	chromosomal	plasmidic	Total
Escherichia coli MG1655	Commensal	4182	0	4182
Escherichia coli O157:H7 sakai	EHEC	5320	104	5424
Escherichia coli O157:H7	EHEC	5265	99	5364
Escherichia coli CFT073	ExPEC	5388	0	5388
Escherichia coli W3110	Commensal	4391	0	4391
Escherichia coli UTI89	ExPEC	5136	166	5302
Escherichia coli 536	ExPEC	4650	0	4650
Escherichia coli APEC O1	ExPEC	4871	423	5294
Escherichia coli S88	ExPEC	4719	137	4856
Escherichia coli UMN026	ExPEC	4860	191	5051
Escherichia coli IAI1	Commensal	4411	0	4411
Escherichia coli ED1A	Commensal	4837	138	4975
Escherichia coli 55989	EAEC	4854	137	4991
Escherichia coli IAI39	ExPEC	4737	0	4737
Escherichia fergusonii	Unknown	4299	57	4356
Shigella boydii Sb227	Shigellosis	4531	184	4715
Shigella dysenteriae Sd197	Shigellosis	4583	514	5097
Shigella flexneri 2a 311	Shigellosis	4351	320	4671
Shigella flexneri 2a2457T	Shigellosis	4380	0	4380
Shigella flexneri 5 str. 8401	Shigellosis	4336	0	4336
Shigella sonnei Ss046	Shigellosis	4638	312	4950
total	-	98739	2782	101521

3. Supplementary results

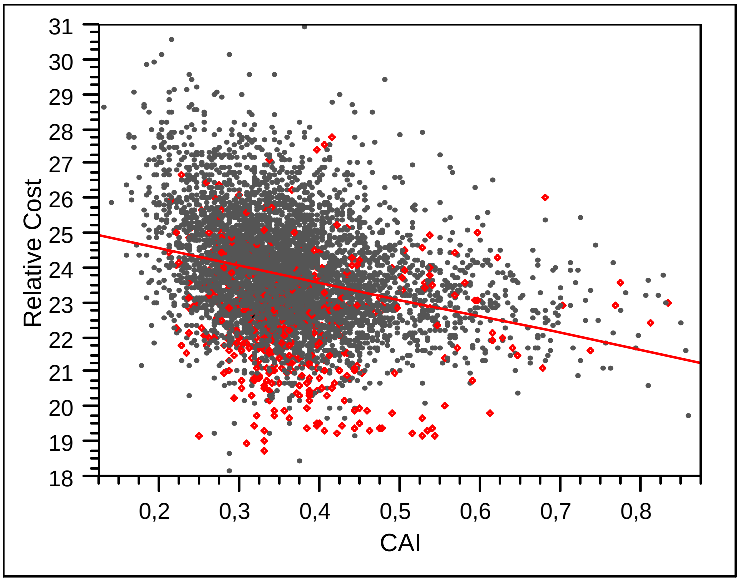

Figure S1. Association between CAI, a proxy of gene expression levels, and protein cost. The regression has a very low R^2=0.088 ($p<0.001$), and the non-parametric Spearman correlation is also low (=-0.3). Red dots correspond to secreted and outer membrane proteins. All points were used in the regression, the colour is just intended to emphasise the biased distribution of relative cost among external proteins. We used the Codon Adaptation Index (CAI) as proxy of gene expression [15]. For this we used ribosomal proteins as the set of highly expressed genes from which optimal codon usage was taken. We then used it to compute CAI on all genes. CAI has been shown to correlate as well with transcriptome data as the latter between themselves and correlates better with proteome data than transcriptome data does [16].

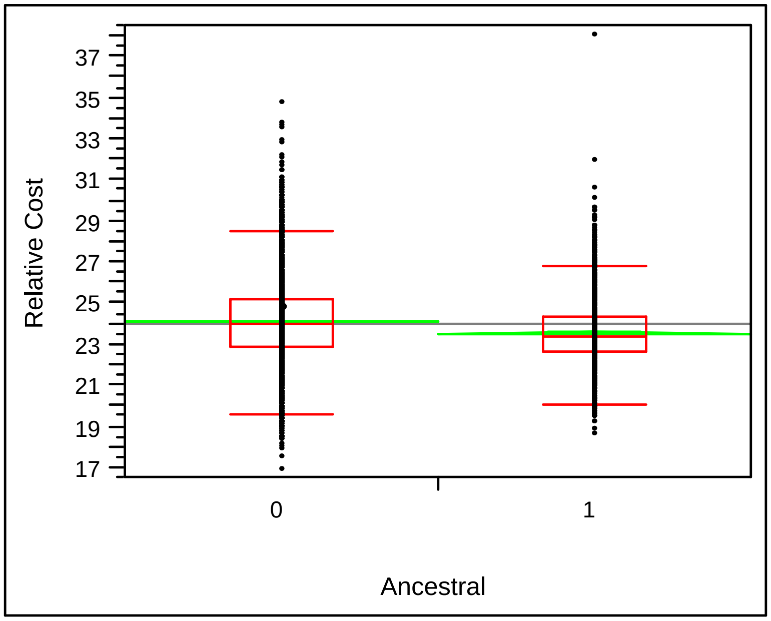

Figure S2. Ancestral proteins (label 1) are less expensive than non-ancestral proteins (label 0) (23.6 versus 23.9 equivalent P, p<0.001, Wilcoxon test). However, in a stepwise regression of protein cost in function of %G+C and the ancestral character of the gene, the first is significant, whereas the second is not (p>0.3). This means that the higher cost of recent proteins stems from their lower G+C content.

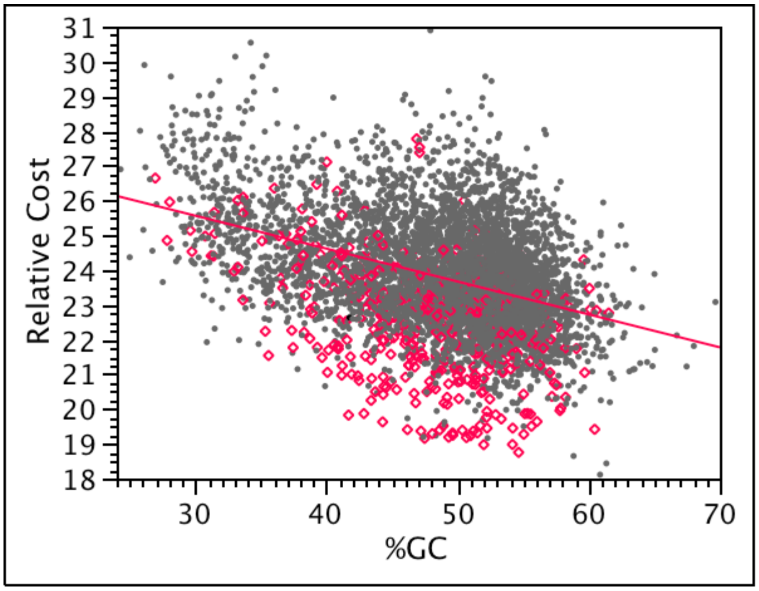

Figure S3. GC richer genes code for less expensive proteins (Relative Cost = 28.406479 - 0.0947468*%GC, R^2=0.16, p< 0.001). Red points correspond to secreted and outer membrane proteins.

4. References

1. Hamilton, W.D. (1964). The genetical evolution of social behaviour. I. J Theor Biol *7*, 1-16.
2. Hamilton, W.D. (1964). The genetical evolution of social behaviour. II. J Theor Biol *7*, 17-52.
3. Lehmann, L., and Keller, L. (2006). The evolution of cooperation and altruism--a general framework and a classification of models. J Evol Biol *19*, 1365-1376.
4. Rousset, F., and Ronce, O. (2004). Inclusive fitness for traits affecting metapopulation demography. Theor Popul Biol *65*, 127-141.
5. Taylor, P.D. (1992). Altruism in viscous populations - an inclusive fitness model. Evol Ecol *6*, 352-356.
6. Gardner, A., and West, S.A. (2006). Demography, altruism, and the benefits of budding. J Evol Biol *19*, 1707-1716.
7. Lehmann, L., Feldman, M.W., and Foster, K.R. (2008). Cultural transmission can inhibit the evolution of altruistic helping. . American Naturalist *172*, 12-24.
8. Grafen, A. (1985). A geometric view of relatedness. Oxford Surveys in Evolutionary Biology *2*, 28-90.
9. McCallum, H., Barlow, N., and Hone, J. (2001). How should pathogen transmission be modelled? TREE *16*, 295-300.
10. Keeling, M.J., and Rohani, P. (2007). Modeling Infectious Diseases in Humans and Animals, (Princeton University Press).
11. Sachs, J.L., Mueller, U.G., Wilcox, T.P., and Bull, J.J. (2004). The evolution of cooperation. Q Rev Biol *79*, 135-160.
12. Smith, J. (2001). The social evolution of bacterial pathogenesis. Proc Biol Sci *268*, 61-69.
13. Brown, S.P., Le Chat, L., De Paepe, M., and Taddei, F. (2006). Ecology of microbial invasions: amplification allows virus carriers to invade more rapidly when rare. Curr Biol *16*, 2048-2052.
14. Touchon, M., Hoede, C., Tenaillon, O., Barbe, V., Baeriswyl, S., Bidet, P., Bingen, E., Bonacorsi, S., Bouchier, C., Bouvet, O., et al. (2009). Organised genome dynamics in the Escherichia coli species results in highly diverse adaptive paths. PLoS Genet *5*, e1000344.
15. Sharp, P.M., and Li, W.H. (1987). The codon Adaptation Index - a measure of directional synonymous codon usage bias, and its potential applications. Nucleic Acids Res *15*, 1281-1295.
16. Coghlan, A., and Wolfe, K.H. (2000). Relationship of codon bias to mRNA concentration and protein length in Saccharomyces cerevisiae. Yeast *16*, 1131-1145.
